# Potential for Local Fertilization: A Benthocosm Test of Long-Term and Short-Term Effects of Mussel Excretion on the Plankton

**DOI:** 10.1371/journal.pone.0156411

**Published:** 2016-06-01

**Authors:** Mehdi Cherif, Monica Granados, Sean Duffy, Pauline Robert, Bérangère Péquin, Vani Mohit, Christopher W. McKindsey, Philippe Archambault, Bruno Myrand, Connie Lovejoy, Réjean Tremblay, Stéphane Plourde, Gregor F. Fussmann

**Affiliations:** 1 Umeå universitet, Institution för Ekologi, Miljö och Geovetenskap, Linnaeus väg 6 SE-901 87 Umeå, Sweden; 2 McGill University, Department of Biology, 1205 ave Docteur-Penfield, H3A1B1 Montréal, Québec, Canada; 3 Institut des sciences de la mer de Rimouski, Université du Québec à Rimouski, 310 allée des Ursulines, G5L3A1, Rimouski, Québec, Canada; 4 Université Laval, Département de Biologie, 1045 av. De la médecine, G1V0A6 Québec, Québec, Canada; 5 Maurice Lamontagne Institute, Fisheries and Oceans Canada, G5H3Z4 Mont-Joli, Québec, Canada; 6 Merinov, 107–125 chemin du Parc, G4T1B3 Cap-aux-Meules, Québec, Canada; Universite Pierre et Marie Curie, FRANCE

## Abstract

Mussel aquaculture has expanded worldwide and it is important to assess its impact on the water column and the planktonic food web to determine the sustainability of farming practices. Mussel farming may affect the planktonic food web indirectly by excreting bioavailable nutrients in the water column (a short-term effect) or by increasing nutrient effluxes from biodeposit-enriched sediments (a long-term effect). We tested both of these indirect effects in a lagoon by using plankton-enclosing benthocosms that were placed on the bottom of a shallow lagoon either inside of a mussel farm or at reference sites with no history of aquaculture. At each site, half of the benthocosms were enriched with seawater that had held mussels (excretion treatment), the other half received non-enriched seawater as a control treatment. We monitored nutrients ([PO_4_^3-^] and [NH_4_^+^]), dissolved oxygen and plankton components (bacteria, the phytoplankton and the zooplankton) over 5 days. We found a significant relationship between long-term accumulation of mussel biodeposits in sediments, water-column nutrient concentrations and plankton growth. Effects of mussel excretion were not detected, too weak to be significant given the spatial and temporal variability observed in the lagoon. Effects of mussels on the water column are thus likely to be coupled to benthic processes in such semi-enclosed water bodies.

## Introduction

Aquaculture, and bivalve farming in particular, has seen explosive growth worldwide [1, 2]. Among the reasons behind this expansion are fishery collapses and increasing human demand for high-quality protein, but also because bivalve aquaculture is generally considered a low-impact activity, in comparison to caged fed aquaculture [3, 4]. Some even envision it as a mitigation measure against eutrophication [5]. However, in order to maximize production, high abundances of filter feeders, including mussels, are suspended in the water column where they graze on natural plankton. This practice may alter the food web around the farms, with potential detrimental consequences [6].

Negative impacts of suspended mussel culture on the benthic fauna have been well documented [7, 8]. Organic matter enrichment results from the accumulation of biodeposits (faeces and pseudofaeces) on the seafloor around mussel farms [9]. Decomposition of this organic matter increases nutrient effluxes from the sediments to the water-column. Eventually, oxygen levels may decline and cause hypoxia [7, 10]. Mussels also affect planktonic communities through predation and competition. Mussels graze directly on the plankton [11, 12] and compete with the zooplankton for the smaller plankton [13]. At large densities, mussels may have a negative feedback on their own growth if they graze on seston at a greater rate than it is replaced by flushing and *in situ* production [14, 15]. The addition of mussels to the water column for cultivation may thus have negative consequences not only on benthic but also on water column communities. However, the same byproducts that alter benthic sediments and communities may also have growth-enhancing effects on plankton communities if they are recycled. Mussels may thus have indirect positive effects on plankton community production in two ways:

1. Long-term enrichment of the seafloor (on the timescale of years) with biodeposits that result in a larger nutrient efflux from the sediments to the water column.
2. Short-term excretion in the water column of readily bioavailable metabolic by-products that fuel the growth of the bacterio- and phytoplankton (on the timescale of minutes to days).

Assessment of the degree to which these processes operate *in situ* could contribute to the establishment of an ecosystem-based aquaculture with the aim to mitigate or prevent the negative effects on seafloors of long-term biodeposit accumulation, while preserving any positive effects of nutrient recycling on the growth of planktonic communities [16]. A first step towards such ecosystem-based aquaculture requires understanding the potential for stimulation of plankton growth by mussel aquaculture.

In this study, we conducted a benthic mesocosm (or benthocosm) experiment to determine the long- and short-term consequences of mussel aquaculture on plankton growth. The experiment was done in Havre-Aux-Maisons Lagoon, Iles de la Madeleine (Québec, Canada), a restricted, oligotrophic lagoon. We placed benthic mesocosms in a mussel farm and in reference sites to highlight the indirect, long-term effects of mussel biodeposition on water-column nutrients and the plankton community. We delivered daily infusions of water containing mussel excreta and un-enriched seawater from control basins to the benthocosms at both farm and reference sites. Comparison between the two treatments evaluates the direct effects of mussel excretion on the planktonic community, isolated from the effects of mussel grazing.

## Materials and Methods

### Site description

**Fig 1:**
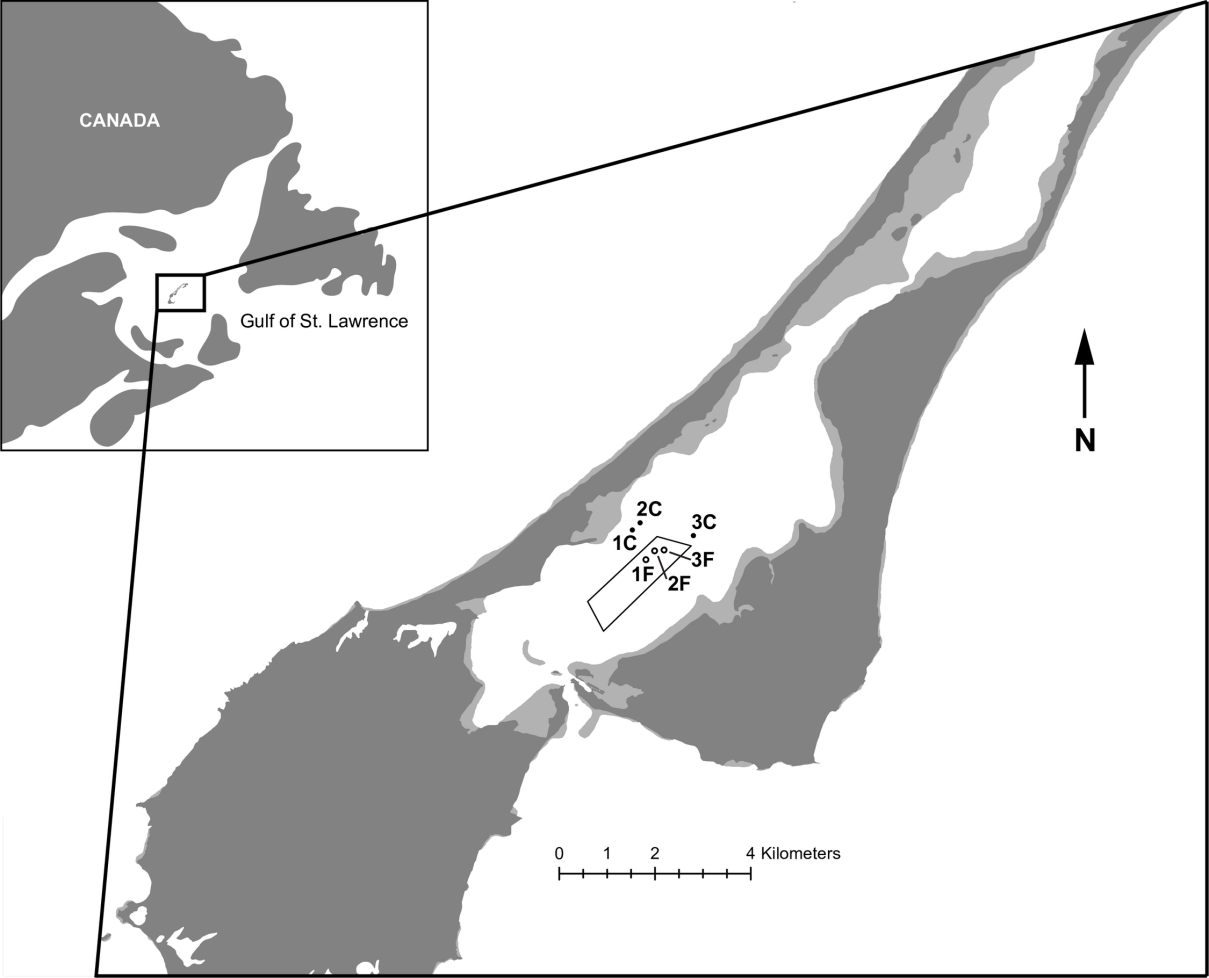
Map and location coordinates of the experimental sites in Iles de la Madeleine (Québec), eastern Canada. (Farm site 1F: 47°25.754' N, 61°49.105’ W; Reference site 1C: 47°26.089' N, 61°49.328’ W; 2F: 47°25.85' N, 61°48.965’ W; 2C: 47°26.168' N, 61°49.196’ W; 3F: 47°25.858' N, 61°48.807’ W; 3C: 47°26.009' N, 61°48.321’ W). The bathymetry outlines are from Énergie et Ressources naturelles Québec and the outline of the Canadian coast line from Statistics Canada.

Havre-aux-Maisons Lagoon (HAM) in Iles de la Madeleine, Québec, Canada (Fig 1) is a shallow lagoon with a maximum depth of 6 m, and a surface area of 30 km^2^, of which approximately 5% is covered by bivalve farm operations [17]. It is classified as a restricted coastal lagoon [18] with only two connections for water exchange; one a restricted tidal inlet connected to the Gulf of St. Lawrence, the second a narrow channel connected to Grande-Entrée Lagoon (Fig 1). Because of the low tidal amplitude and frequent high winds that are a defining characteristic of Iles de la Madeleine, water column mixing is mainly wind driven [19]. Water residence time varies between 25 to 45 days in the whole lagoon, and 25–30 days in the farm sites, depending on the strength of prevailing winds [19]. Nutrient concentrations and phytoplankton biomass are characteristically low throughout the summer in the lagoon with some evidence for nitrogen limitation [20, 21], hence its classification as oligotrophic [22, 23]. Secchi depths are generally between 2–3 m in August, signaling low to moderate turbidity (1997–1998 data from the Observatoire Global du Saint-Laurent).

### Benthocosms

Experiments to test the influence of mussels on the water column were done in and around a small mussel farm in HAM (active since the 1980s). This work was conducted with a Fisheries and Oceans Canada Research Notice (IML-2010-47). For work done within the mussel farm, we had written permission from Michel Fournier, the mussel farm owner. Between August 14 and August 18, 2010, we deployed twelve open-bottom, 556 L, transparent (87% light transmission) fiberglass cylinders (Solar Components Corporation), henceforth referred to as benthocosms.

**Fig 2:**
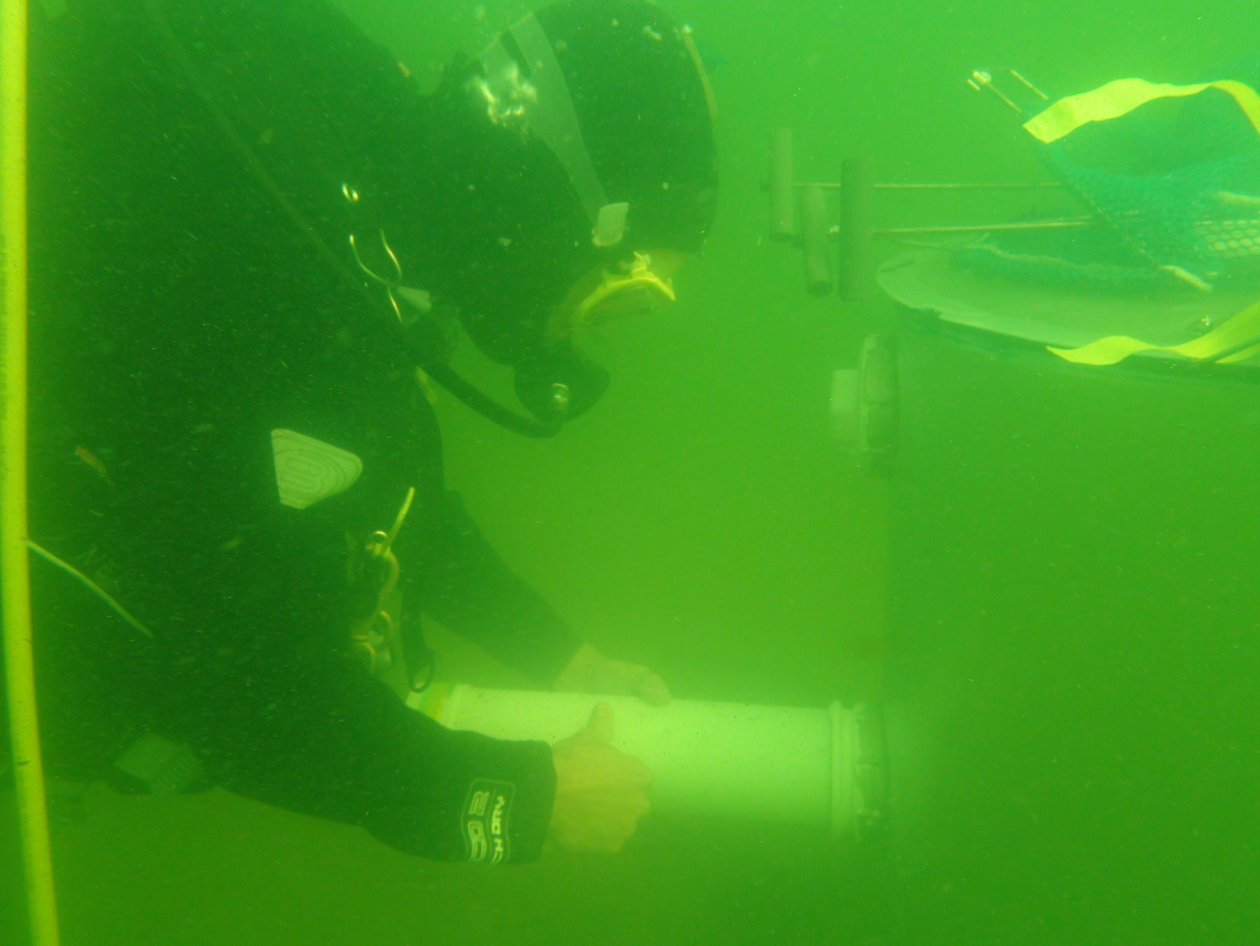
Picture taken on the first day of the experiment, just after the insertion of a benthocosm in the sediment, showing a diver sampling for nutrients and plankton. Photo courtesy of Simon Bourgeois.

We selected the western-most portion of the farm for the experiments, which contained mussels in their third year (i.e. market size). This portion of the farm was selected for its proximity to the areas of the lagoon with no history of aquaculture. Three sites within the mussel farm were selected for the “farm” substrate treatment of the experiment. Three other sites with no history of aquaculture were selected as reference sites (Fig 1). Increasing the distance of reference sites from the mussel farm to ensure they are independent was traded off with maximizing their abiotic similarities to farm sites. We settled on a distance of 650–680 m between farm-reference sites, paired in a block design. The average water depth in all selected sites was ca. 6 m. Just prior to setting up the benthocosms, nutrient and plankton samples were collected from all sites and analyzed according to methods described further below. The open-bottomed benthocosms were inserted into the sediment to a depth of 30 cm (giving 500 l of water). Each benthocosm was fitted with 6 sampling ports of 10.2 cm diameters vertically distributed along opposite sides (3 per side) of each benthocosm to facilitate sampling without disturbing the setup. Water samples from each benthocosm were obtained daily using a 3 L sampling syringe designed to join precisely with the sampling ports of the benthocosm. Two paired benthocosms were deployed at each farm and reference site, one benthocosm receiving nutrient infusions and the other control infusions. Nutrient infusions were prepared by placing 60 mussels in opaque incubation container containing18 L of filtered (54 μm) seawater for a period of 24 hours. Control infusions without mussels were prepared simultaneously in identical incubation containers with 18 L of filtered (54 μm) seawater. The biomass of mussels in the incubations was set to be the same as the biomass of mussels in the nearby farm on a per-volume-of-water basis. Water from the incubation containers was transferred to portable containers on boats. Benthocosms were visited daily by scuba divers using portable diving bags and the sampling syringe system. On each sampling occasion, nutrient infusion water was injected into the benthocosms by a scuba diver, following sample collections for plankton and nutrients (Fig 2). A nitex screen covering an opened port on the top of the mesocosm was used to allow water without plankton to flow in and out so as to not create pressure differentials on the sediment. Oxygen data were also taken using a YSI 6600 EDS probe with an optical oxygen sensor (mg l^-1^ ± 0.01).

### Sampling

#### Nutrients

Samples for nutrient analyses were collected from the 3 L water samples collected from all the ports of the benthocosms. Ammonium (in μM) was measured only at the start and end of the experiment immediately after sampling, following the OPA (orthophtaldialdhehyde) method of Holmes et al [24] using a 10-AU Turner Designs fluorometer.

The remaining water samples were filtered on 0.8 μm GF/F syringe filters and frozen (-80°C) in Falcon tubes. Total reactive phosphorus and nitrate + nitrite (in μM) were determined for the frozen samples using standard protocols [25] adapted for the AA3 Autoanalyzer (SEAL Analytical Inc), with a detection limit of 0.08 and 0.1 μM respectively. Most values for nitrate + nitrite were near or below the detection limit, with substantial variation between replicate analyses on sub-samples. Consequently, nitrate + nitrite was not further considered in this study (see Table 1).

**Table 1:** Mean values±SD (sample size) for all the variables measured in the mesocosms during the experiment.

	Farm sites (sample size)		Reference sites (sample size)		Total (sample size)
Input	-	+	-	+	
Variable:					
**PO**_**4**_^**3-**^ **(μM)**	3.5±0.73 (**15**)	3.6±1.19 (**15**)	1.6±0.15 (**15**)	1.5±0.12 (**15**)	2.6±1.22 (**60**)
**NH4**^**+**^ **(μM)**	12.0±1.74 (**6**)	8.3±5.17 (**6**)	2.0±1.36 (**6**)	1.8±0.83 (**6**)	6.0±5.15 (**24**)
**NO3+NO2 (μM)**	1.7±2.32 (**15**)	1.2±0.17 (**15**)	1.0±0.05 (**15**)	1.1±0.06 (**15**)	1.3±1.16 (**60**)
**DO (mg.L**^**-1**^**)**	6.9±2.32 (**6**)	5.7±1.55 (**6**)	7.1±0.91 (**6**)	7.2±0.90 (**6**)	6.7±1.55 (**24**)
**Chl a (μg. L**^**-1**^**)**	4.9±1.06 (**12**)	5.2±1.58 (**12**)	6.0±1.26 (**12**)	6.0±1.15 (**12**)	5.5±1.33 (**48**)
**totPhyt (#.ml**^**-1**^**)**	2.6e5±0.74 (**12**)	2.4e5±0.57 (**12**)	2.3e5±0.58 (**12**)	2.2e5±0.32 (**12**)	2.4e5±0.58 (**48**)
**totBact (#.ml**^**-1**^**)**	11.3e6±1.78 (**12**)	11.2e6±2.86 (**12**)	8.0e6±0.93 (**12**)	7.7e6±1.46 (**12**)	9.6e6±2.54 (**48**)
**totAdult (#.L**^**-1**^**)**	19±19.14 (**6**)	15.8±16.87 (**6**)	43.1±26.78 (**6**)	48.1±23.23 (**6**)	31.5±25.04 (**24**)
**totLarv (#.L**^**-1**^**)**	10.8±16.04 (**6**)	3.3±3.64 (**6**)	25.3±8.29 (**6**)	42.6±33.25 (**6**)	20.5±23.42 (**24**)

#### Pico- and nanoplankton (phytoplankton plus free bacteria)

Daily samples for chlorophyll *a*, eukaryotic and bacterial cell counts, were taken from the top, middle and bottom ports. The water was mixed and then filtered through a 64 μm-diameter net to remove the zooplankton. Chlorophyll *a* concentrations were estimated from 250 mL of water filtered onto 25 mm diameter GF/F glass fiber filters (Whatman). Samples were stored at -80°C until analyzed. Chlorophyll *a* was extracted using the hot ethanol method [26, 27] with a Cary Eclipse Spectrofluorometer. Prokaryotic and eukaryotic cell counts were done using flow cytometry (FCM). Samples were first fixed in the dark with 1% (final concentration) glutaraldehyde and stored at -80°C until analysis with and without addition of the fluorescent dye SYBR Green I (Invitrogen). FCM counts were performed on an Epics Altra Flow Cytometer (Beckman Coulter) and data were analyzed using the Expo32 v1.2b software (Beckman Coulter) to quantify pico- (0.2–2 μm) and nano- (2–20 μm) chlorophyll containing cells, and heterotrophic bacteria with low nucleic acid (LNA) or high nucleic acid (HNA) [28].

#### Zooplankton

The zooplankton was sampled at the start and end of the experiment. 6 L of water were removed from the top, middle and bottom ports of each benthocosm and filtered through a 64 μm sieve. The collected organisms were preserved in 75% ethanol for enumeration and identification. The organisms were identified to genera and species, where possible, under a dissection microscope.

### Statistical analyses

#### Explanatory variables

The two manipulated factors detailed below were crossed and the factorial design was repeated in three blocks (Fig 1):

(i) location (Loc) of the benthocosms under the mussel farm (F) or in an adjacent historically unfarmed area of the lagoon (C), acting as reference sites; (ii) the type of water injected daily into the benthocosms (Input): water from mussel infusions (+) and water from control incubations without mussels (-). The combination of factors was repeated in 3 blocks (block factor with three modalities: “1”, “2”, and “3”). There was no within-block replication, yielding a total of 12 benthocosms.

Sampling was done every day from the start (Aug. 14^th^) to the end of the experiment (Aug. 18^th^). Sampling occasion was coded as a covariable, SamplN. Some variables were measured only at the start and end of the experiment ([NH_4_^+^], zooplankton counts), while others were measured daily from the start of the experiment ([PO_4_^3-^], flow cytometer cell counts), and some measured starting one day after the start of the experiment (chlorophyll a, total phytoplankton and total bacterial abundances). Thus sampling of the different variables was done on 2, 4 or 5 days. Injection of water infusions was done daily, just after sampling.

#### Response variables

Effects of location (Loc), injection treatment (Input), time (SamplN) and spatial heterogeneity (Block) on nutrient concentrations ([PO_4_^3-^] and [NH_4_^+^]), dissolved oxygen (DO), chlorophyll a (chla), total phytoplankton abundances (totPhyt) and total bacterial abundances (totBact) were analyzed statistically using the nlme package (version 3.1–103) of the R software (Rstudio version 0.96.330). Plots of replicate analyses on nutrient samples suggested that measured nutrient concentrations ([PO_4_^3-^] and [NH_4_^+^]) were log-normally distributed (data not shown) and thus data for those variables were log-transformed prior to statistical analyses. Total abundances of larvae and adult zooplankton (totLarv and totAdult respectively) were recorded as count data, with many zero occurrences, and assumed to follow a negative binomial distribution. Thus, a negative binomial regression model was fitted to the data with all possible interactions between Loc, Input and SamplN, using the glm.nb function from the MASS package (version 7.3–17) in R. Random effects of blocks were subsequently tested on the residuals from the negative binomial regression. The dataset used for the statistical analyses are summarily presented in Table 1 and can be found online at http://dx.doi.org/10.5281/zenodo.16336.

S1 Appendix in Supporting Information explains the methods used for the statistical analyses in detail. S2 Appendix includes the R code of all the analyses performed. We only briefly describe some of the analyses here:

Linear mixed models were applied to the data, with sampling occasion (SamplN) as a covariable, location and mussel infusion as fixed factors, and blocks as random factors. We then used a model selection procedure to reach the most parsimonious and likely model, starting from the full linear mixed model (including all potential interaction terms and random effects, Eq 1).

$$y_{ijkl}=(\gamma_{0}+\gamma_{i}+\gamma_{j}+\gamma_{ij}+c_{k}+c_{ik}+c_{ijk})+(\beta_{0}+\beta_{i}+\beta_{j}+\beta_{ij}+b_{k}+b_{ik}+b_{ijk}).SamplN_{l}+(\alpha_{0}+\alpha_{i}+\alpha_{j}+\alpha_{ij}+a_{k}+a_{ik}+a_{ijk})SamplN_{l}{}^{2}+\varepsilon_{ijkl}$$ (1)

where i is the index for Loc (C or F), j is the index for Input (+ or–mussel water), k is the index for block (1,2 or 3), l is the index for SamplN (0,1,2,3 or 4), **γ**, β, α are fixed effect coefficients and c, b, a are random effect coefficients.

Terms were dropped one by one, starting with random terms and then fixed terms, to yield simpler, nested models, which were compared to the full model using log-likelihood ratio tests or the AIC criterion.

Finally, normality and homoscedasticity of the residuals from the selected model were visually inspected using QQ-plots and residual-vs-fitted scatter plots.

Multivariate analyses. In order to investigate the effects of location (Loc), injection treatment (Input), time (SamplN) and spatial heterogeneity (Block) on planktonic community compositions, we generated site redundancy analysis ordinations (RDA) for both the pico- and nanoplankton and zooplankton data, based on Eqs 2 and 3:

Y∼block+loc*input*SamplN (2)

Redundancy analysis model for the pico- and nanoplankton data where Y denotes the composition data.

Y∼block+loc*input (3)

Redundancy analysis model for the zooplankton data where Y denotes the composition data.

We produced ordinations for start and end compositions for the zooplankton data to reduce the number of factors displayed. We subsequently performed a Procrustes analyses on the reference and farm ordinations for the zooplankton data to generate residuals for each paired reference and farm site, Procrustes analysis compares two ordinations by rotating one ordination and its set of points around a second fixed ordination to minimize the squared distance between points [29].

## Results

**Fig 3:**
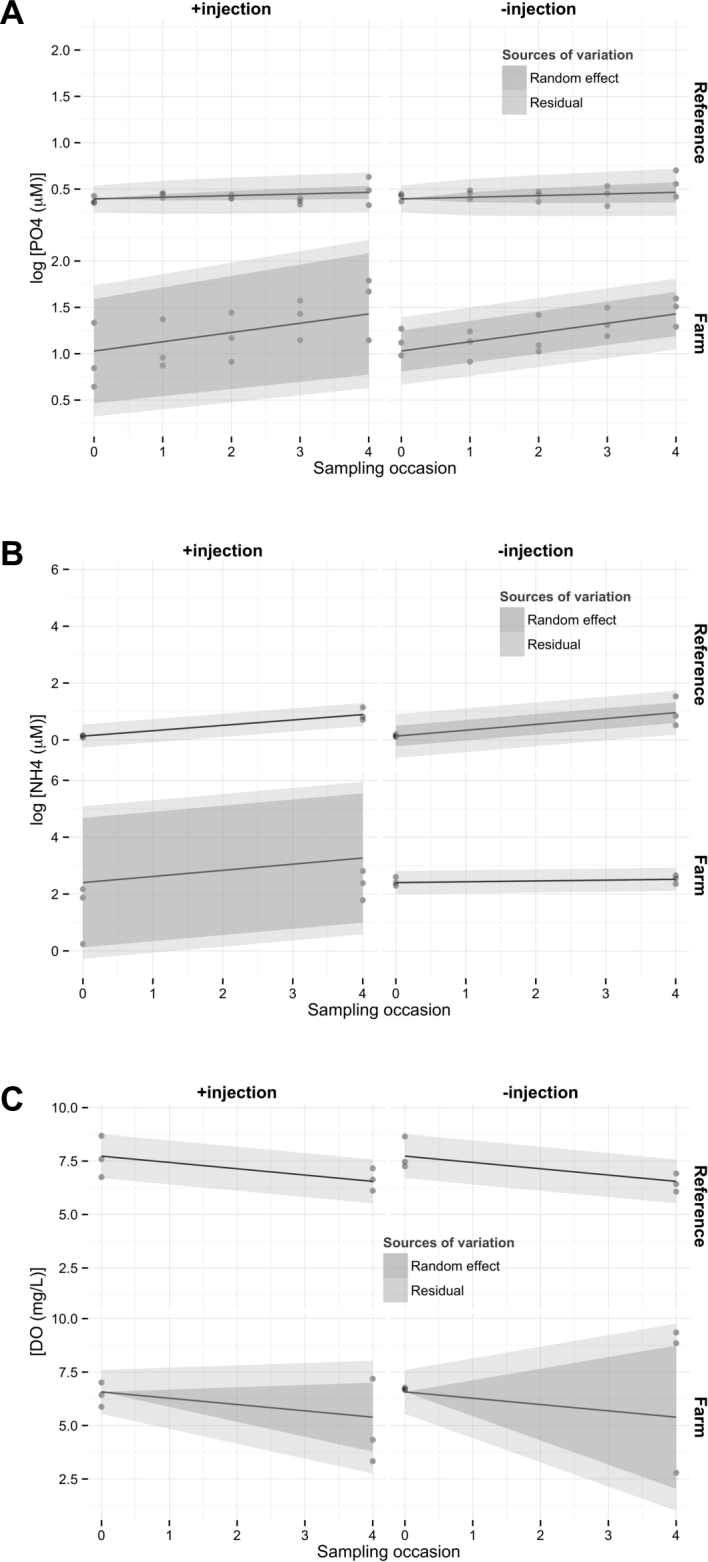
log PO_4_ and B) log NH_4_ as a function of sampling occasion (SamplN). A) Predictions from the selected models (regression lines) are reported on the figure. Areas represent 2 times the standard error due to random differences between blocks (Random effects, dark grey) or 2 times the residual errors (light grey).

**Fig 4:**
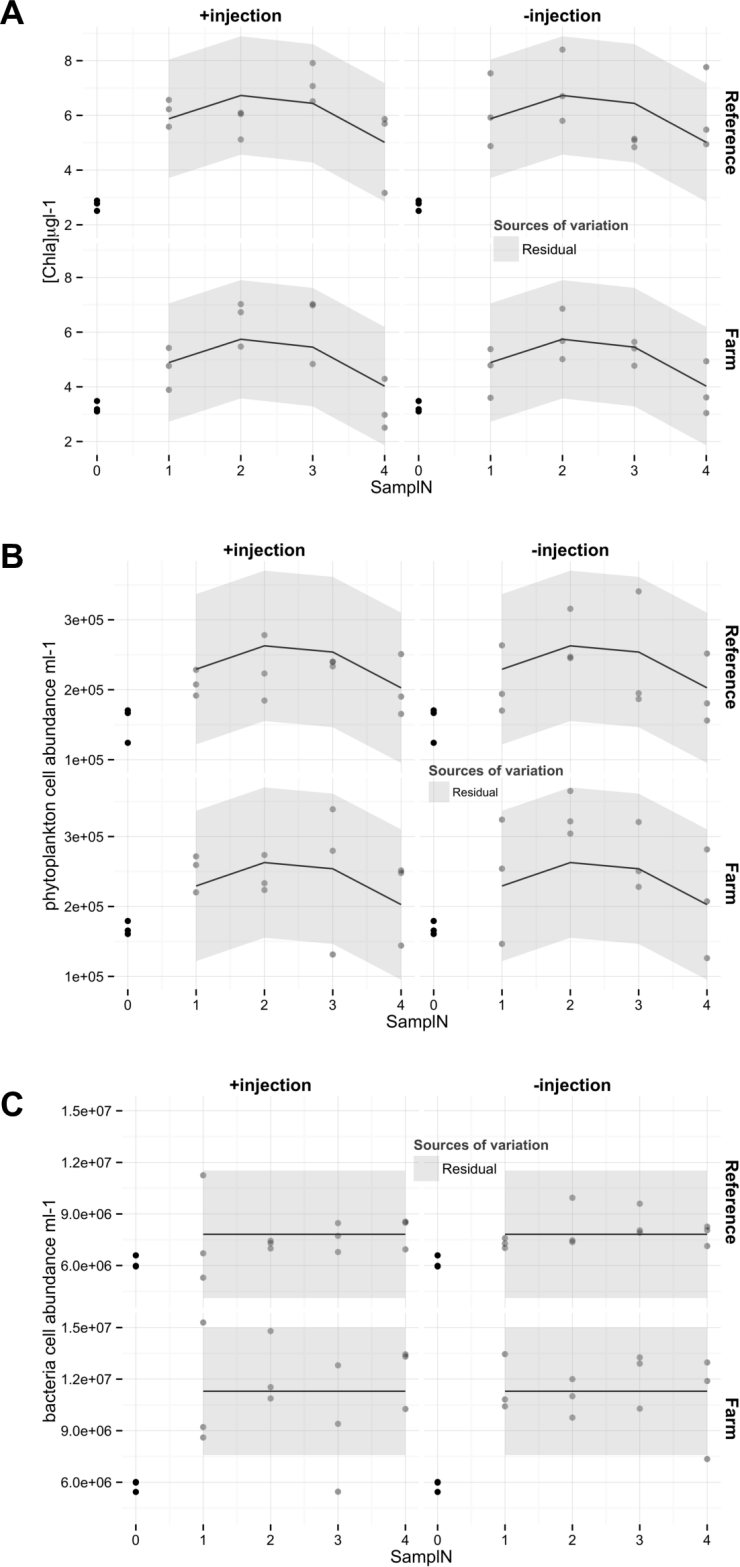
A) Chla B) Phytoplankton abundance, and C) Bacteria cell abundance as a function of sampling occasion (SamplN). Predictions from the selected models (regression lines) are reported on the figure. Areas represent 2 times the residual errors (light grey). Random effects were not significant. For total phytoplankton and bacteria abundances, the initial samples of the mussel injection factor (Input) modalities were pooled together (black dots). Hence, they were not included in the model analysis.

**Fig 5:**
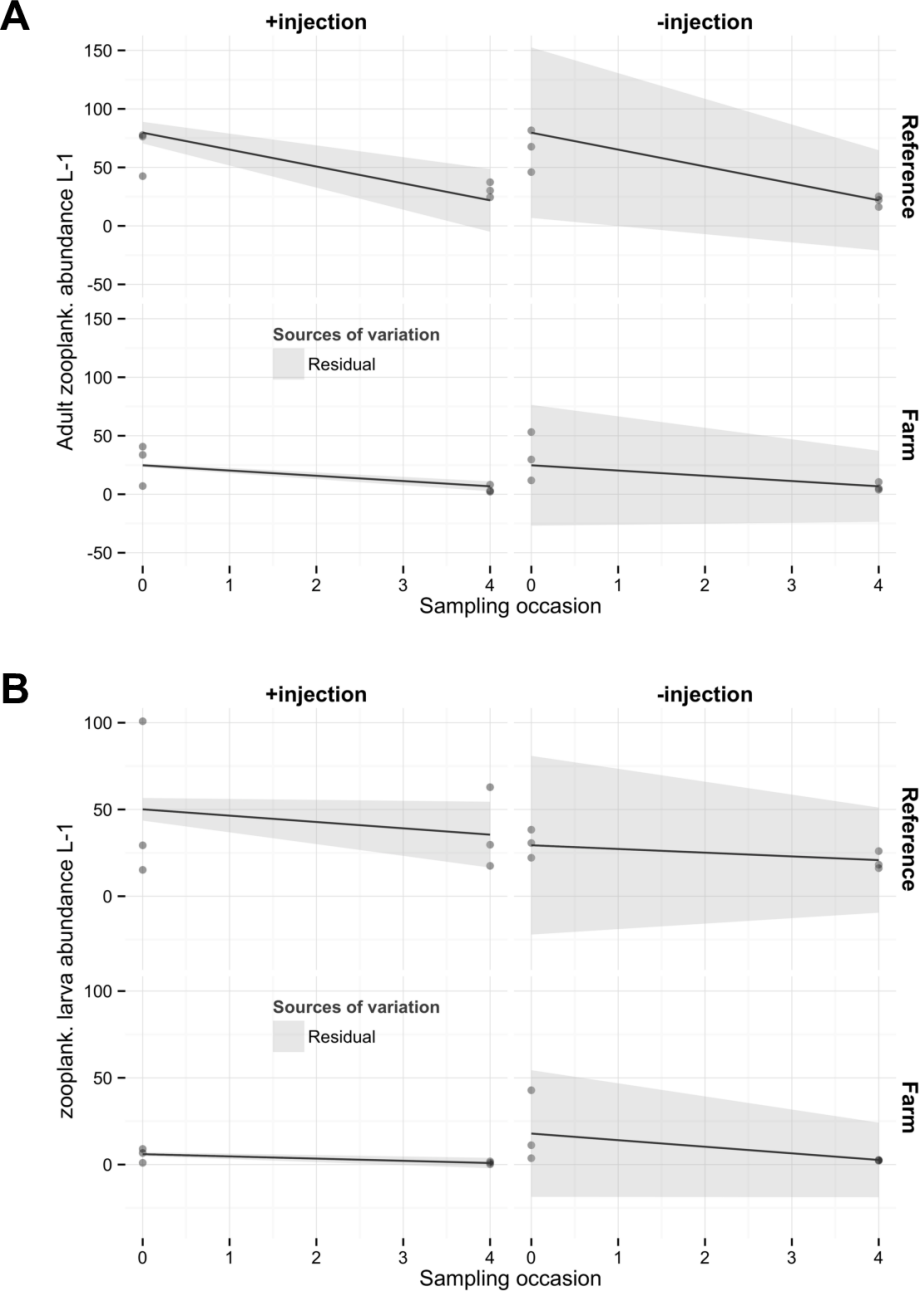
A) Adult zooplankton counts and B) zooplankton larva counts as a function of sampling occasion (SamplN). Predictions from the selected models (regression lines) are reported on the figure. Areas represent 2 times the residual errors (light grey). Random effects were not significant.

Location affected both initial logPO4 levels (parameter γ_i_, Table 2, p<0.0001; Fig 3A), and their response to incubation (parameter β_i_, Table 2, p = 0.0011; Fig 3A). Injection of mussel infusion (Input factor) did not affect mean logPO4 values significantly (no β_j_ parameter retained in Table 2, Fig 3A). Variation between blocks (random effects) was clearly different between distinct combinations of location and input treatments (parameters c_ijk_ and b_ijk_, Table 2). Figs 3–5 show that random effects were strongest in the benthocosms placed in farm locations and receiving mussel infusions (F+).

**Table 2:** Selected statistical models for the univariate response variables.

Variable	Selected model:			
	intercept	Slope	Quadratic term	error
***logPO4***	*γ*_*0*_*+ γ*_*i*_ *+c*_*ijk*_	*(β*_*0*_*+ β*_*i*_ *+b*_*ijk*_*) SampleN*_*l*_		
***logNH4***	*γ*_*0*_*+γ*_*i*_ *+c*_*ijk*_	*(β*_*0*_ *+ β*_*ij*_*) SampleN*_*l*_		
***DO***	*γ*_*0*_*+γ*_*i*_	*(β*_*0*_ *+b*_*ijk*_*) SampleN*_*l*_		
***Chla***	*γ*_*0*_*+γ*_*i*_	*(β*_*0*_*) SampleN*_*l*_	*(α*_*0*_*) SampleN*_*l*_^*2*^	*+ε*_*ijkl*_
***totPhyt***	*γ*_*0*_	*(β*_*0*_*) SampleN*_*l*_	*(α*_*0*_*) SampleN*_*l*_^*2*^	
***totBact***	*γ*_*0*_*+γ*_*i*_			
***log(totLarv)***	*γ*_*0*_*+γ*_*i*_*+γ*_*j*_ *+γ*_*ij*_	*(β*_*0*_*+ β*_*i*_*) SampleN*_*l*_		
***log(totAdult)***	*γ*_*0*_*+γ*_*i*_	*(β*_*0*_*) SampleN*_*l*_		

Notations: **γ**, β, α = fixed effects; a, b, c = random effects; indices: 0 = overall intercept, slope or quadratic term; i = location (“F” or “C”); j = input (“+” or “-“); k = block (“1”, “2” or “3”); l = Sampling occasion (0 to 4).

LogNH4 increased significantly between the start and the end of the experiment (Fig 3B, parameter β_0_, Table 2, p = 0.0006). Initial conditions were clearly different between locations (parameter γ_i_, Table 2, p<10^−9^). Similar to logPO4, benthocosms in farm sites receiving mussel infusions (F+) showed large significant differences among blocks (leading to the retention of the random parameter c_ijk_ in the model, Table 2). Reference benthocosms that received control infusions also showed significant differences between blocks, but to a lesser extent. The average slopes, indicating change of logNH4 over the duration of the experiment, were similar between locations, but not when separated according to Input (parameter β_ij_, Table 2, p = 0.0315). DO measurements were initially different between reference and farm sites (lower in the latter, Fig 3C, parameter γ_i_, Table 2, p = 0.0298). They decreased in parallel in both sites during the experiment (parameter β_0_, Table 2, p = 0.0009), but with significantly larger differences between blocks in farm sites, particularly those not receiving mussel injections (Fig 3C, parameter b_ijk_ in Table 2).

Initial samples from the two mussel-injection treatments for Chla, totPhyt and totBact (Sampling occasion 0) were pooled together, thus precluding their inclusion in the mixed linear model analysis. Only the data from the second day of sampling (Sampling occasion 1) could be used as input for the linear mixed effect model. However, a nonparametric Wilcoxon test comparing the bulk values from the initial sampling occasion (SamplN = 0) to the first sampling occasion (SamplN = 1) shows a significant increase in Chla concentrations (p = 0.0001, Fig 4A). Starting from first sampling occasion (SamplN = 1), Chla follows a quadratic increase through time (α_0_, Table 2, p = 0.0006). Among the fixed factors, only location had a significant effect on Chla initial conditions (γ_i_, Table 2, p = 0.003). Moreover, despite an obvious scattering of the data, no systematic differences in chlorophyll a among blocks were found.

Similarly, total phytoplankton abundances increased between sampling occasions 0 and 1 (Wilcoxon, p-value = 0.005). The temporal response is also quadratic (α_0_, Table 2, p = 0.02, Fig 4B), but contrary to Chla, effects of location were not retained within the selected model.

Total bacteria abundances increased between sampling occasions 0 and 1 (Wilcoxon, p-value = 0.003) but did not vary thereafter (Fig 4C). Abundances on sampling occasion 1 differed significantly between locations, with a 70% increase of abundances in farm sites, compared to reference sites (γ_i_, Table 2, p = 5.10^−8^).

Larval and adult zooplankton counts both declined dramatically during the incubation (around 40 and 70% declines, respectively, Fig 5A and 5B). No significant random effects on the residuals from the full-factorial negative binomial regression model were found (Corrected log-likelihood tests, p = 0.99 and p = 0.66 respectively).

**Fig 6:**
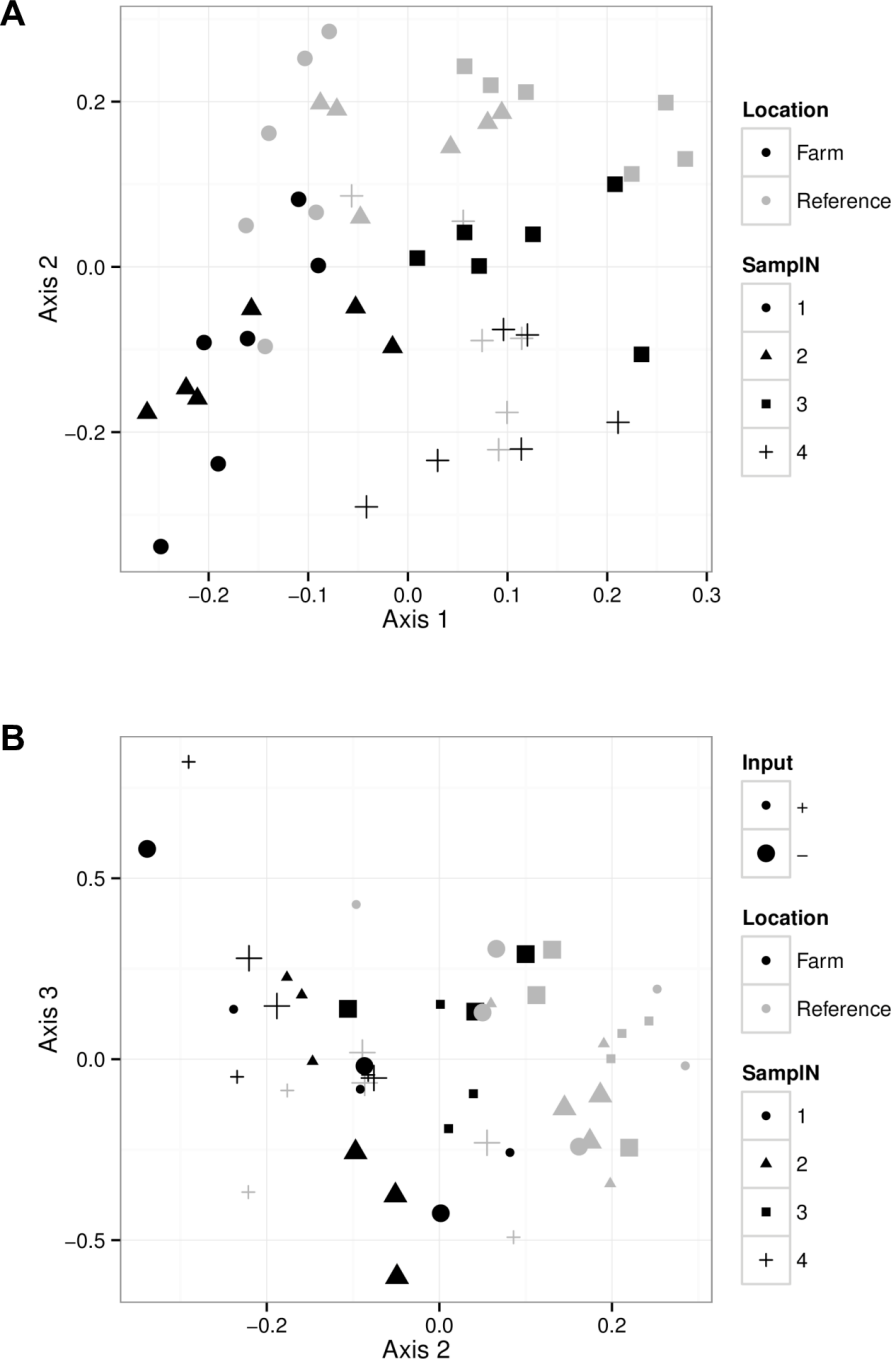
Redundancy analysis (RDA) of the microplankton community group composition as a function of the experimental factors. A) The first axis is associated with sampling occasion. Location is associated with the second axis. B) Input is only significantly associated with the 3rd axis.

RDA of the pico- and nanoplankton community composition as a function of the explanatory variables (Location, Input and blocks treated as fixed effects) yielded 3 significant principal component axes (permutation test for redundancy analysis, p_1_ = 0.001, p_2_ = 0.001 and p_3_ = 0.009 respectively). The first axis is mainly linked to SamplN and captures the changes in population abundances through time (78% of variation) (Fig 6A). Bacteria (High Nucleic Acid-containing–HNA, and Low Nucleic acid bacteria–LNA) exhibited the most variation through time. The second axis is mainly the variation associated with location and end date of the experiment (3.8% of the variation). The third axis is associated with Input in interaction with SamplN and Location, but explains only 1.7% of the variation (Fig 6B).

**Fig 7:**
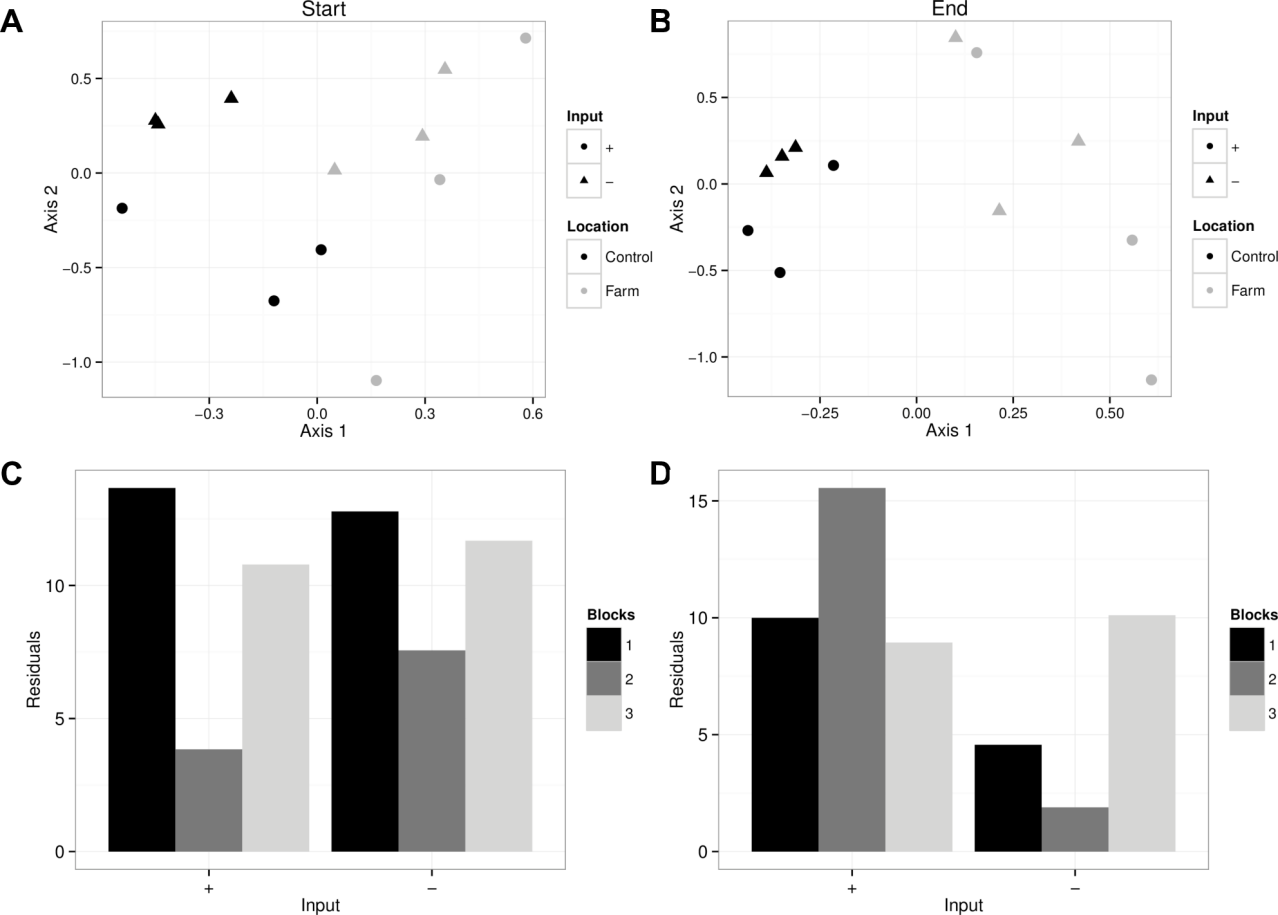
Redundancy analysis (RDA) of the zooplankton community composition at A) the start and B) end of the experiment. In both cases, the first axis is associated with location and is the only significant axis. B and D show the residuals from a Procrustes analysis comparing farm sites to reference sites at the start and end of the experiment respectively, showing no apparent difference in residuals related to the mussel injection treatment (- and + inputs).

Only the first axis of the zooplankton community RDA explains a significant portion of variation (30.7%; permutation test, p = 0.001). It is mainly associated with the Location factor (Fig 7). This highlights the difference in the zooplankton community composition between farm and reference sites, with, e.g., foraminiferidae, and harpacticoida copepods found in greater abundance in farm sites, and *Acartia* and *Oithona* copepods found mainly in reference sites.

## Discussion

The influence of mussel farming on the environment is often seen through the lens of its effects on the benthic environment [30–32]. Eutrophication and associated degradation of functional and species richness have been documented for the benthos, even at moderate densities of mussel farming [33, 34]. Although a number of studies have evaluated effects of mussel farming on nutrient fluxes [15, 35], few have investigated their effects on plankton communities [36] (but see [37]). Our study is also one of few studies that report on potential indirect effects of bivalves on the zooplankton (see also Fig 4 in [36]). Other direct effects are expected and have been otherwise well documented: the biomass of seston components that are within the size spectrum of the mussels’ filtration apparatus are likely to be reduced in mussel farms [37], although in our experiment, we only observed a significant decrease in the abundance of the zooplankton.

In our study we were concerned with two indirect effects by which suspended mussels may increase nutrient availability in the water column and thereby “fertilize their own garden.” The first effect is through the remobilization of nutrients previously accumulated in biodeposits under mussel farms. The second effect is through the excretion of nutrient-rich metabolic by-products by mussels directly into the water column. Our experiment endeavored to capture and tease apart the two types of indirect effects:

1. Differences between the benthocosms under the mussel farm and those in reference sites capture the long-term effects of biodeposition on nutrients and planktonic food web components.
2. Differences between the dynamics and end point of the planktonic food web components in the benthocosms receiving mussel infusion injections and those receiving ambient seawater are indicators of short-term effects of mussel excretion.

Long-term effects of biodeposits proved particularly strong in our experiment, affecting initial nutrient concentrations ([PO_4_^3-^] and [NH_4_^+^]), Chla, total bacteria and zooplankton abundances, as well as the dynamics of nutrients and the zooplankton in the benthocosms.

In contrast, short-term effects of mussel excretion were negligible, or, when marginally significant, potentially an artifact of the high initial variability among sites under the mussel farm. The concentrations of nutrients in the mussel injections were set to emulate the mean levels of nutrients produced daily in the mussel farm. Hence, we conclude that the excretion of metabolic by-products by mussels in the farm used in our experiment (HAM) is unlikely to affect the water column food web on the time scale of a few days.

### Long-term effects of mussel farms on the water column

Before the start of the experiment, farm sites had higher concentrations of nutrients ([PO_4_^3-^] and [NH_4_^+^]) and bacteria abundances but lower levels of Chl *a* and zooplankton abundances. In terms of community composition, phytoplankton communities were very similar between the two locations whereas zooplankton communities were strikingly different.

These contrasting properties of the two locations are consistent with what is expected from long-term biodeposition impacts on sediments: farm sites generally show signs of eutrophication and of organic matter enrichment such as increased nutrient fluxes from the sediments [3, 7].

The similar phytoplankton abundances and community compositions between farm and reference locations at the start of the experiment suggest that mussel grazing impacts on the phytoplankton are averaged over the whole lagoon through water mixing [38, 39].

We propose three mechanisms for the lower initial zooplankton abundances at farm sites: i) Resource competition between mussels and the zooplankton for the phytoplankton [40]. However, since phytoplankton abundances were similar between farm and reference sites, this mechanism can be ruled out; ii) Active avoidance of farm sites by the zooplankton; and iii) Zooplankton predation by mussels [12]. Mussels can ingest particles up to 1000 μm [41], including several zooplankton species that fall within this size range [42]. Indeed, it has been suggested that zooplankton consumption above mussel beds could be great enough to significantly reduce populations of nauplii and copepodites [43].

Initial bacterial abundances were slightly higher in farm sites, suggesting mussels do not readily consume bacteria in the lagoon [21]. Other case studies have shown decreased bacteria abundances above mussel beds [43] whereas a meta-analysis of aquaculture effects on the water column indicate no significant effects of bivalves on bacteria [36]. Thus, it seems that more detailed studies of mussel farming impacts on the bacterioplankton are necessary.

Beyond initial conditions, long-term biodeposition also affected the temporal responses of nutrient concentrations and of zooplankton abundances. The rate of increase of PO_4_^3-^ concentrations through time was higher in farm sites, probably a combined result of confinement within benthocosms and higher PO_4_^3-^ effluxes from sediments under mussel farms [8]. Rates of increase of [NH_4_^+^] were significantly lower in farm sites receiving control water infusions (F-) than those receiving nutrient infusions. We note, however, that the first sites also started with higher initial NH_4_^+^ concentrations. So, one might envision a ceiling NH_4_^+^ concentration that was reached sooner in F- benthocosms. The faster decline of zooplankton in farm sites could be explained by hypoxia: dissolved oxygen levels measured on the last day in some farm benthocosms were just a few mg/L, indicative of potential hypoxia.

### Short-term, mussel excretion effects

The initial abundances of zooplankton larvae differed significantly between benthocosms that received injections of mussel infusions and those receiving control water (Fig 4B). Given that the initial sampling took place before first injection, and that the difference is largely due to one count of >600 larvae in one benthocosm (C+, block 6, Fig 4B), no biological meaning can be inferred from this result.

The pico- and nanoplankton community composition also varied as a function of mussel nutrient infusion status (3^rd^ principal component axis in the RDA analysis), but only marginally (this axis accounts for only ca. 1.7% of total variation).

In summary, the short-term effects of mussel excretion in this experiment were either too small to be detected or were confounded by variability in initial conditions. The absence of any obvious effects of bioavailable nutrient additions through mussel water injections may be due to nutrient concentrations in mussel infusions being too low to elicit a response in the plankton. Although PO_4_^3-^ concentrations in mussel infusions were on average 10 times higher than those of control water (means of 7.2 μM and 0.7 μM respectively), 3 L of water (nutrient-enriched or control) was added to each 500 L benthocosm each day, increasing PO_4_^3-^ concentration within benthocosms by about 0.04 μM L^-1^ Day^-1^, a modest increase when compared to background effluxes from sediments previously measured in a nearby location (around 10 and 60 μmol m^-2^ h^-1^ in a reference site and in a 2-year-old blue mussel farm, respectively) [17]. Increase in NH_4_^+^ concentrations in benthocosms with mussel infusions was greater, since mussel excretion increased NH_4_^+^ concentrations more than PO_4_^3-^. However, this increase was apparently not sufficient to enhance the growth of the plankton above that observed in benthocosms with control water. However, factors other than [PO_4_^3-^] and [NH_4_^+^] may have limited plankton growth at the time of the experiment (see below).

The density of mussels used to prepare water infusions was calculated to simulate the mean rate of nutrient addition from the mussel lines in the local mussel farm. In 2010, 136 mussel lines were present in the farm in an area slightly larger than 150 ha, with each line measuring 250 m and supporting ca. 300 mussels per m (François Bourque, MAPAQ, personal communication). Since these additions were too low to elicit an increase in the growth of the phytoplankton, it is likely that the current densities of mussel lines in HAM also have a limited or no effect on plankton growth through excretion on the time scale of a few days (5 days in the case of our experiment).

### Temporal responses and effects of containment

All parameters measured varied over time: [NH_4_^+^] and [PO_4_^3-^] reached higher values at the end of the experiment compared to initial conditions (Fig 2A and 2B). The zooplankton decreased in abundance. Variables related to the phytoplankton (Chl *a* concentration and total phytoplankton abundance) showed a unimodal pattern over time, somewhat resembling a bloom pattern (Fig 3A and 3B). However, different phytoplankton groups showed different patterns [39]. Some groups decreased throughout the experiment (picoeukaryotes), while others increased exponentially (nanocyanobacteria). Bacteria abundances rose rapidly to be greater by the second sampling occasion and then stayed at this level.

That most pico- and nanoplanktonic groups responded to manipulation fairly similarly under all treatments suggests that a limiting factor, other than those manipulated in the experiment, was lifted when the benthocosms were placed *in situ* (see [43] on effects of containment). Hence, the growth patterns observed were probably, to a large extent, unrelated to the effect of mussels.

Chl *a* measurements taken in and around the farm site at the same time as the benthocosms were in place suggest no coincident bloom occurred outside of the benthocosms for the pico- and nanoplankton as a group [44]. However, the abundances of the bacterioplankton and of some phytoplankton groups followed similar patterns inside and outside, while picoeukaryotes decreased within the benthocosms and increased outside of them [44]. Which factor(s) limited the growth of the various pico- and nanoplankton groups is thus unclear, although we noticed that water temperature started increasing on Aug. 15^th^, around the same time as those groups that had parallel trajectories inside and outside the benthocosms started increasing in abundance [44].

### Effects of spatial heterogeneity

Only inorganic concentrations ([PO_4_^3-^], [NH_4_^+^] and DO) varied as a function of blocks in a consistent way. Overall, it seems that spatial heterogeneity most affected nutrients but that its effect was buffered at higher trophic levels, probably reflecting: i) the longer time scale of growth processes in comparison to nutrient-linked processes (e.g., cell effluxes and uptakes) and ii) the homogenizing effect of water mixing on initial conditions. Simultaneous measurements of Chla and phytoplankton abundances near our sites, before, during and after the experiment confirmed the role of wind mixing in the homogenization of plankton abundance between farm and reference sites [39].

Water mixing strength is demonstrably an important factor in setting nutrient levels and carrying capacities in aquaculture [44]. The lagoon in which the experiment was conducted has been characterized as a “restricted” lagoon, one where water and sediment exchanges with the ocean are reduced compared to a leaky lagoon [18]. However, water mixing within the lagoon is significant due to strong and frequent winds [45], which are sufficient to influence the attachment strength of mussel byssus in the farm [46]. The hydrological properties of the lagoon would have contributed to the starting conditions for the experiment by effects of: i) the localized accumulation of sediments under farm sites [33]; and ii) the homogeneity in planktonic components across the farm and reference sites at the start of the experiment. Hence, whether mussels in farms can enhance the growth of their own living resources through short-term nutrient recycling seems, to a large extent, to be a function of the whole-lagoon hydrodynamic conditions. Short-term recycling is less likely to be important in well-mixed habitats. Testing such a hypothesis will require experimental approaches at a larger scale than that possible using benthocosms. Mesocosms typically cut the enclosed waters from fluctuations and material exchanges with the surroundings [47]. More complicated mesocosms have been designed to circumvent this limitation [48] and may better simulate *in situ* conditions, but must be traded off with increased logistic costs [49]. Hence, our benthocosm experiment is more accurately a test for the potential response of the water-column to mussel excretion in absence of water exchange. This is still interesting information, a measure of the immediate, localized response of the water-column as affected by short-term and long-term mussel excretion.

In conclusion, addition of water enriched by mussel excretions to levels similar to those expected to be produced by mussel lines in the HAM farm did not increase the biomass of the plankton species that would contribute to the mussel’s diet. However, there was a clear effect of long-term biodeposition from mussels on plankton growth that may potentially feedback positively on mussel growth. Similar investigations about mussel’s indirect effects on plankton communities should be extended to other hydrodynamic regimes for both farmed and natural populations of mussels.

## Supporting Information

S1 Appendix Statistical procedure for the analysis of the benthocosm data. (DOCX)

S2 Appendix R code for the implementation of the statistical procedure for the analysis of the benthocosm data. (R)
