# Supplementary material for: Potential for Local Fertilization: A Benthocosm Test of Long-Term and Short-Term Effects of Mussel Excretion on the Plankton
Source: PLoS One. 2016 Jun 1;11(6):e0156411. doi: 10.1371/journal.pone.0156411 (PMC4889037; doi:10.1371/journal.pone.0156411)
Supplement: S1 Appendix — (DOCX) [file pone.0156411.s001.docx]

**Statistical procedure for the analysis of the benthocosm data:**

The analyses went through 4 or 5 steps, depending on the response variable:

1. The structure of the regression between the variable of interest and sampling occasion (SamplN) was chosen by fitting and comparing linear, pure quadratic and quadratic regressions for data in each mesocosm unit separately, using the lmList function (equation 1). This step was applied in the case of variables measuring members of the plankton community, in order to detect possible bloom patterns. A linear model was used by default for the inorganic variables logPO4, logNH4 and DO (the last two measured only at the beginning and the end of the experiment).

Equation 1: regression model within each mesocosm

y_ijkl_= **γ**_ijk_+ β_ijk_ SamplN_l_ + α_ijk_ SamplN_l_^2^+ ε_ijkl_

i index for Loc (C or F)

j index for Input (+ or – mussel water)

k index for block (1,2 or 3)

l index for SamplN (0,1,2,3 or 4)

1. Afterwards, one linear mixed model is fitted (equation 2), with sampling occasion (SamplN) as a covariate using the regression structure selected in the previous step. Loc (parameters **γ**_i_, β_i_ and α_i_ in box 1), Input (parameters **γ**_j_, β_j_ and α_j_) and their interactions (parameters **γ**_ij_, β_ij_ and α_ij_) were chosen as fixed factors that can potentially affect the intercept, the slope and the acceleration of the regression. Random factors related to blocks are considered to act on 3 levels: blocks can affect the mean intercept, slope and acceleration (block effects c_k_, b_k_ and a_k_), they can affect the regression parameters differently in different locations (block-within-location effects c_ik_, b_ik_ and a_ik_) and finally, within locations, the regression parameters might vary between Input modalities (block-within-input-within-location effects c_ijk_, b_ijk_ and a_ijk_).

Equation 2: Full linear mixed model (linear and quadratic components included or not depending on model significance from equation 1)

y_ijkl_=(**γ**_0_+ **γ**_i_+ **γ** _j_+ **γ**_ij_+c_k_+c_ik_+c_ijk_) + (β_0_+β_i_+β_j_+β_ij_+b_k_+b_ik_+b_ijk_) SamplN_l_ +(α_0_+α_i_+α_j_+α_ij_+a_k_+a_ik_+a_ijk_) SamplN_l_^2^+ε_ijkl_

c_k_~ N (0,σ^2^_γ0_) b_k_~N(0,σ^2^_β0_) a_k_~ N (0,σ^2^_α0_)

c_ik_~ N (0,σ^2^_γi_) b_ik_~N(0,σ^2^_βi_) a_ik_~ N (0,σ^2^_αi_)

c_ijk_~ N (0,σ^2^_γij_) b_ijk_~N(0,σ^2^_βij_) a_ijk_~ N (0,σ^2^_αij_)

ε_ijkl_~ N (0,σ^2^)

i index for Loc (C or F)

j index for Input (+ or – mussel water)

k index for block (1,2 or 3)

l index for SamplN (0,1,2,3 or 4)

1. Obviously, the full linear mixed effect model in equation 2 is over-parameterized. A model reduction procedure is then followed by comparing the full model (equation 2) to models with one or more random effects dropped, using a corrected log-likelihood ratio test ([Verbeke & Molenberghs 2000](#_ENREF_8)). The model selected is the model with the simplest random effect structure that is not significantly different from the full model (i.e., as likely as the full model at a 0.05 significance level) and who is more parsimonious than the full model (i.e., has a smaller BIC value).
2. Once the structure of the random effects is chosen, the significance of the fixed effect terms is tested using F-ratio tests and all non-significant fixed effect terms (p-value>0.05) are dropped from the model. Finally, a linear mixed effect model, including both the selected random effect structure and the significant fixed effect terms is compared to the model with all the fixed effect terms using a corrected log-likelihood ratio test.
3. In a final step, the validity of the selected model is tested by inspecting the distribution of the residuals using QQ-plots and residual-vs-fitted scatter plots. If the residuals are obviously correlated to the fitted values, heteroscedastic, or non-normally distributed, terms dropped during the model selection process are sequentially reintroduced, until a satisfactory residual distribution is reached.

Total densities of larvae and adult zooplankton (all species lumped together) are count data, with many zero occurrences. They are thus likely to follow a negative binomial distribution. Effects of factors on these two response variables (totAdult and totLarv) was analyzed in 3 steps:

1. A negative binomial regression model was fitted to the data with all possible interactions between Loc, Input and SamplN, using the glm.nb function from the MASS package (version 7.3-17). The significance of the various effects are tested using the same procedure as in steps 4 and 5 of linear mixed effect models.

Equation 3: Negative binomial regression model used for the total density of adult and larvae zooplankton

log(y_ijkl_)=(**γ**_0_+ **γ**_i_+ **γ** _j_+ **γ**_ij_) + (β_0_+β_i_+β_j_+β_ij_) SamplN_l_ +ε_ijkl_

ε_ijkl_~ NB(0,σ^2^, η)

i index for Loc (C or F)

j index for Input (+ or – mussel water infusion)

k index for block (1, 2 or 3)

l index for SamplN (0 or 1)

1. The negative binomial regression model does not accommodate random factors. Hence, to test for potential effects of blocks, we conducted a-posteriori analysis of the residuals from the regression. A pure random effect model was fitted to the standardized Pearson residuals (equation 4). A reduced model was then selected using the same procedure as in step 3 for the mixed effect linear models.

Equation 4: Random effect linear model used to test the effects of block on the residuals from the negative binomial linear regression.

y_ijkl_=(c_k_+c_ik_+c_ijk_) + (b_k_+b_ik_+b_ijk_) SamplN_l_+ε_ijkl_

c_k_~ N (0,σ^2^_γ0_) b_k_~N(0,σ^2^_β0_)

c_ik_~ N (0,σ^2^_γi_) b_ik_~N(0,σ^2^_βi_)

c_ijk_~ N (0,σ^2^_γij_) b_ijk_~N(0,σ^2^_βij_)

ε_ijkl_~ N (0,σ^2^)

i index for Loc (C or F)

j index for Input (+ or – mussel water infusion)

k index for block (1, 2 or 3)

l index for SamplN (0,1,2,3 or 4)

The microplankton (phytoplankton+ bacteria) and zooplankton community compositions were analyzed using a Redundancy analysis (RDA, vegan package version 2.0-4).

A model with all the explanatory variables (equation 5) is fitted to the table of the taxonomic group distribution across mesocosm units, after counts were transformed using a Hellinger transformation. Significance of the principal component axes is then tested using a bootstrapping method, as implemented in the anova.cca function.

Equation 5: Structure of the model used in the Redundancy Analysis (RDA)

y_ijklz_= f(**γ**_i_+ **γ** _j_+ **γ**_l_ + **γ**_il_ + **γ**_jl_ + **γ**_ij_ + **γ**_jjl_ + **γ**_k_+ε_ijklz_)

i index for Loc (C or F)

j index for Input (+ or – mussel water)

k index for block (1, 2 or 3)

l index for SamplN (0,1,2,3 or 4)

z index for taxonomic group
